# Sarcomatous Transformation of a Medically Treated Lactotroph Pituitary Neuroendocrine Tumor?

**DOI:** 10.1007/s12022-023-09757-1

**Published:** 2023-02-24

**Authors:** Merryl Terry, Gerald Reis, Andrew Horvai, Melike Pekmezci, Arie Perry

**Affiliations:** 1 Department of Pathology, University of California San Francisco (UCSF), San Francisco, CA USA; 2 Department of Pathology, Memorial Healthcare System, Hollywood, FL USA; 3 Department of Pathology, Clinical Cancer Genomics Laboratory, UCSF, San Francisco, CA USA; 4 Department of Pathology and Neurological Surgery, UCSF, San Francisco, CA USA

**Keywords:** Pituitary, Adenoma, PitNET, Sarcomatous, Transformation

## Case History

This 49-year-old man presented with headaches, diplopia, and elevated serum prolactin (> 2000 ng/mL). Brain MRI revealed a 4.6 cm enhancing mass in the pituitary fossa, with inferolateral invasion. He was treated medically with cabergoline with normalization of prolactin but no improvement in symptoms. He underwent tumor resection.

## What Is Your Diagnosis?

**Fig. 1:**
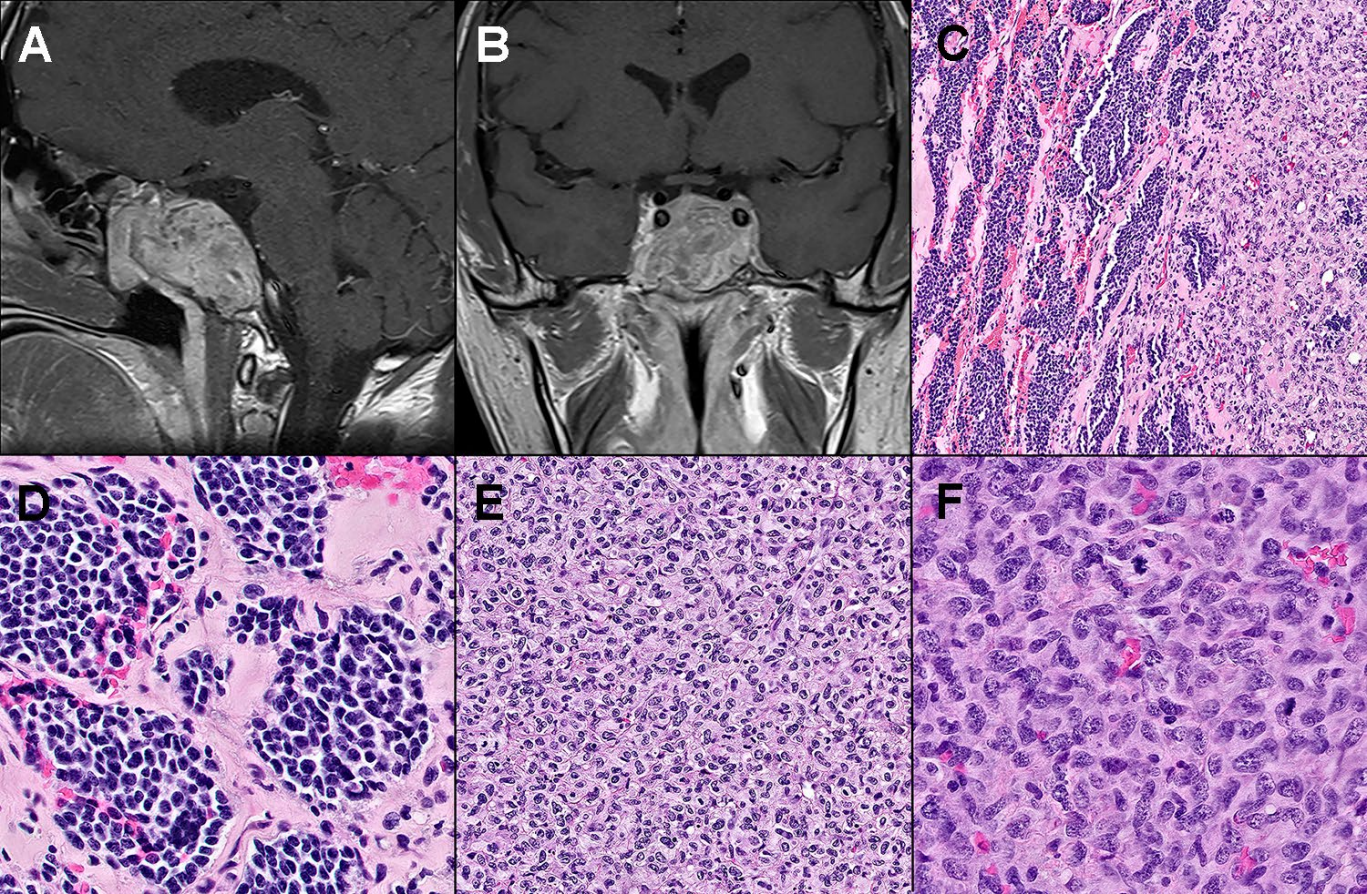
Post-contrast T1-weighted MRI showed an enhancing, invasive sellar mass **A** (sagittal), **B** (coronal). Histology revealed sheets of poorly differentiated cells adjacent to nests of well-differentiated neuroendocrine cells **C**. The PitNET featured small monomorphic cells with high N/C ratios, embedded in a densely collagenous stroma, consistent with dopamine agonist treatment effects **D**. The sarcoma consisted of pleomorphic cells with abundant mitoses **E** and **F**

**Fig. 2:**
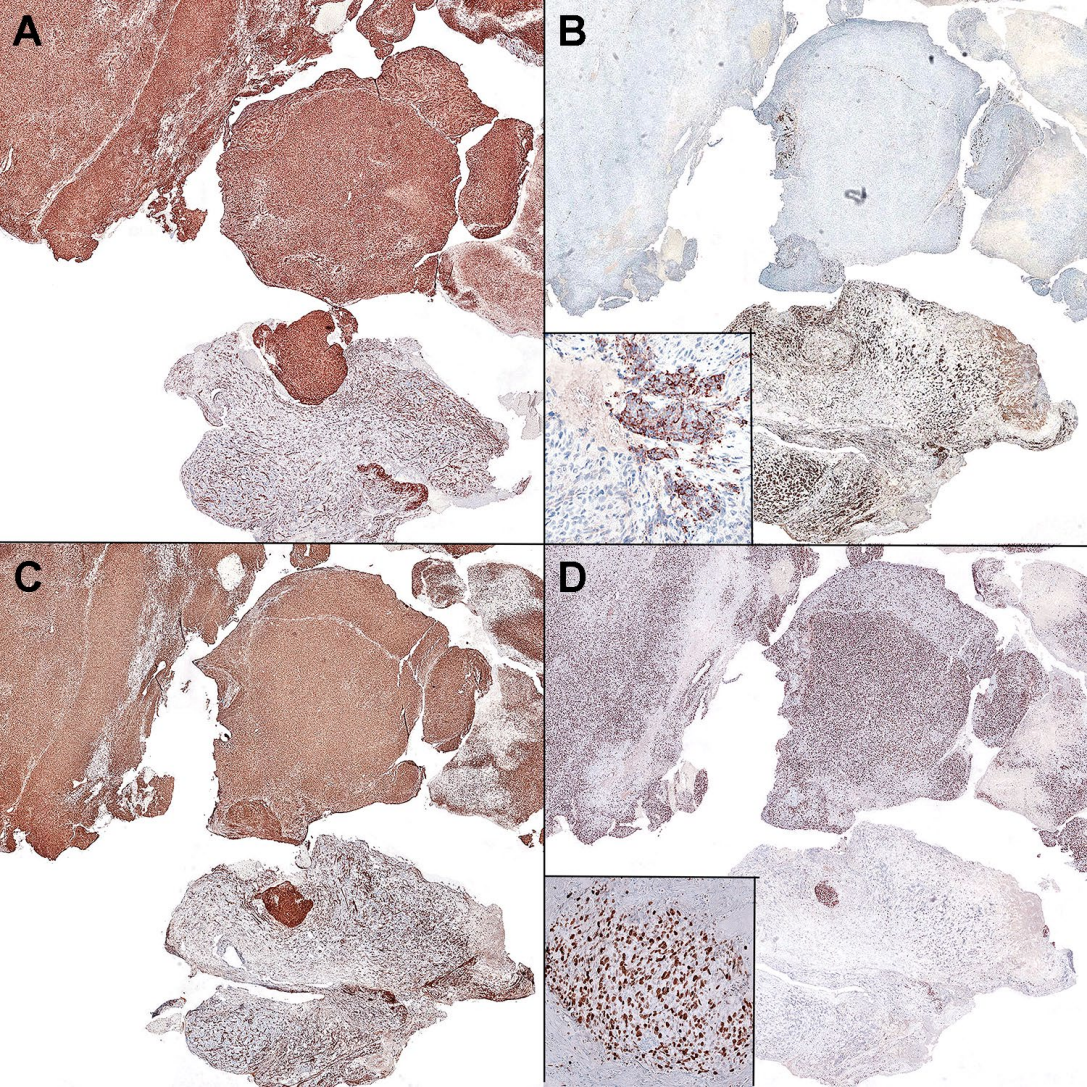
At low magnification, the sarcoma is in the top half, and the PitNET is in the bottom half. The sarcoma was diffusely positive for CD34 **A**. The PitNET was densely granulated, with strong prolactin positivity **B** (inset: higher magnification at the interface). The sarcoma was also positive for vimentin **C**. The Ki-67 labeling index was < 1% in the PitNET and ~ 60% in the sarcoma **D** (inset: higher magnification at the interface)

Figure composites (see Figs. 1 and 2).

## Diagnosis: Densely Granulated Lactotroph Pituitary Neuroendocrine Tumor (PitNET) with Treatment Effects and Adjacent Sarcoma

A biphasic neoplasm was found, with a well-defined but focally intermingled PitNET and a high-grade sarcoma, not otherwise specified (Fig. 1). The PitNET was comprised of small epithelioid cell nests with minimal cytoplasm and delicate “salt and pepper” chromatin. Stromal hyalinization was prominent and in combination with the high N/C ratio was consistent with dopamine agonist treatment effects. The sarcomatous component contained anaplastic spindled to epithelioid cells arranged in sheets and fascicles, with pleomorphic nuclei, open chromatin, scattered macronucleoli, and moderate cytoplasm. There were > 20 mitotic figures per 10 high-power fields. The PitNET was reticulin-poor (though the surrounding fibrotic stroma was positive). Extensive intercellular reticulin characterized the sarcomatous component. By immunohistochemistry (Fig. 2), the densely granulated PitNET cells were positive for prolactin and PIT1, but negative for SF1, TPIT, ACTH, growth hormone, TSH, FSH, and LH. The sarcomatous cells were diffusely positive for vimentin and CD34, but negative for all pituitary hormones and transcription factors, cytokeratin, melanocytic, glial, and muscle markers. The Ki-67 labeling index was < 1% in the PitNET, but up to 60% in the sarcomatous component.

The tumor was further characterized by the UCSF500 Next-Generation Sequencing (NGS) cancer panel. Tumor-only sequencing was performed separately on the two components, revealing an overlapping common activating missense mutation in the *PDGFRB* oncogene in both regions (allele frequency: 8% in PitNET and 60% in sarcoma). No additional alterations were identified in the PitNET, but the sarcomatous component contained an activating missense mutation in *PIK3CA*, a truncating missense mutation in *RECQL4*, biallelic homozygous deletion of *CDKN2A/CDKN2B*, and *TERT* promoter rearrangement. Chromosomal copy number analysis revealed an aneuploid genome in both components but essentially no overlap in the copy number variants. While the possibility of contamination could not be entirely excluded, it was considered unlikely, as the other sarcoma-associated alterations were not identified in the PitNET sequencing.

## Comment

PitNETs are generally well-differentiated NETs with a favorable prognosis, although a subset is associated with considerable morbidity and mortality. Sarcomatous transformation of PitNETs is exceedingly rare and is nearly always associated with prior irradiation [1, 2]. We are only aware of a single additional case reported, similarly showing a sarcoma in association with a lactotroph PitNET treated with bromocriptine 5 years prior to surgery [3]. The sarcoma appeared similar to ours, although the reported Ki-67 labeling index of 7.2% was considerably lower.

The genetic alterations identified in our sarcoma were not specific for a more distinct tumor type [4, 5]. However, given the common molecular alteration between the sarcoma and PitNET, the malignant transformation of a single clonal neoplasm is favored over a collision tumor. Imatinib, used in the treatment of other *PDGFRB*-fusion positive neoplasms (e.g., gastrointestinal stromal tumor and dermatofibrosarcoma protuberans), may theoretically have clinical utility. Our patient experienced dramatic improvement to his headaches and partial improvement to his double vision after surgery. He was recommended to follow up with oncology and was subsequently evaluated for post-surgical radiotherapy.

## Data Availability

Not applicable.
